# The Roles of Exosomal microRNAs in Diffuse Large B-Cell Lymphoma: Diagnosis, Prognosis, Clinical Application, and Biomolecular Mechanisms

**DOI:** 10.3389/fonc.2022.904637

**Published:** 2022-06-02

**Authors:** Somayeh Yazdanparast, Zoufang Huang, Shayan Keramat, Mehrdad Izadirad, Yi-Dong Li, Letao Bo, Ahmad Gharehbaghian, Zhe-Sheng Chen

**Affiliations:** ^1^Department of Hematology and Blood Bank, School of Allied Medical Science, Shahid Beheshti University of Medical Science, Tehran, Iran; ^2^Ganzhou Key Laboratory of Hematology, Department of Hematology, The First Affiliated Hospital of Gannan Medical University, Ganzhou, China; ^3^Department of Hematology and Blood Bank, Faculty of Medicine, Mashhad University of Medical Science, Mashhad, Iran; ^4^Department of Pharmaceutical Sciences, St John’s University, New York, NY, United States

**Keywords:** diffuse large B-cell lymphoma (DLBCL), exosome, miRNAs, diagnosis, prognosis, treatment

## Abstract

**Background:**

Diffuse large B-cell lymphoma (DLBCL) is a heterogeneous neoplasm and is characterized as the most common subtype of non-Hodgkin lymphoma (NHL). Despite 60–70% of all patients being cured with R-CHOP therapeutic regimen (Cyclophosphamide, doxorubicin, vincristine, and prednisone, combined with rituximab), remaining patients display aggressive disease. Therefore, there is an urgent need to develop novel diagnostic, prognostic, and predictive biomarkers. Recently, exosomal miRNAs have been approved as novel biomarkers in DLBCL due to their potential involvement in lymphomagenesis.

**Material and Methods:**

We conducted an investigation on the potential role of exosomal miRNAs as diagnostic, prognostic, and predictive biomarkers in DLBCL in the PubMed, Scopus, and Web of Science search engines. We searched by using a combination of keywords, such as diffuse large B-cell lymphoma, DLBCL, miRNA, microRNA, miR, exosome, exosomes, exosomal, extracellular vesicles, EVs, and secretome. Then, search results were narrowed based on specific inclusion and exclusion criteria.

**Results:**

Twelve articles were eligible for our systematic reviews. Among them, nine discussed diagnostic biomarkers, three considered prognostic significance, four evaluated therapeutic efficacy, two studies were conducted *in vitro*, and three assessed molecular pathways associated with these exosomal miRNAs in DLBCL.

**Discussion:**

According to our systematic review, exosomal miRNAs are not only useful for diagnosis and prognosis in DLBCL but are also promising therapeutic tools and predictors of response to therapy. Although promising results so far, more research is required to develop innovative biomarkers.

## Introduction

Diffuse large B-cell lymphoma (DLBCL) is a heterogeneous group of lymphoma, accounting for approximately 30% of non-Hodgkin lymphoma (NHL) in the United States, which represents the most prevalent subtype of NHLs (1). Currently, the definitive diagnosis of DLBCL requires histopathology. Despite biopsies-based examination allowing detection of enlarged lymph nodes, it is an invasive procedure (2). Although more than 50% of cases with DLBCL are cured with R-CHOP (Cyclophosphamide, doxorubicin, vincristine, and prednisone, combined with rituximab), DLBCL is an aggressive disease in which relapsed or refractory patients have a poor prognosis (3).

Extracellular vesicles (EVs) can be divided into two categories based on their size: small extracellular vesicles (sEVs) and large extracellular vesicles (lEVs). Exosomes in 40–150 nm diameter are a special group of sEVs (4). Exosomes are secreted by diverse cell types, including cancer cells. Exosomes carry many important bioactive molecules including nucleic acids (DNA, mRNA, miRs), protein, and lipids (5). Tumor cell-derived exosomes communicate between exosome-originated cells within the tumor microenvironment (TME), which potentiates tumorigenesis. Hence the cancer-derived exosome is an applicable biomarker for cancer diagnosis, prognosis, and treatment (6). Exosomes influence different aspects of hematological malignancies (7). In lymphoma, exosomes participate in disease progression *via* bone marrow microenvironment modulation, enhancing angiogenic ability, suppressing the immune response, and contributing to drug resistance. Moreover, they can serve as drug delivery tools, and be used for therapeutic response prediction and therapeutic targets (8).

microRNAs (miRNAs) are unique content of cancer cell-derived exosomes and have crucial roles in lymphomagenesis. miRNAs impact lymphoma progression by controlling cell growth, proliferation, differentiation, survival, and apoptosis. Therefore, microRNA expression profiles can serve as prognostic, diagnostic, therapeutic, and predictive biomarkers in lymphoma (9). Of note, miRNAs have unique features including stability, detectability in many biological fluids, and relatively resistance to RNAase degradation, making them useful biomarkers (10, 11).

The high prevalence of DLBCL among NHLs and the challenges in diagnosis and treatment of this disease require the development of new approaches to manage this disease. Considering the key roles of exosomal miRNAs in lymphoma-associated molecular mechanisms can direct our attention to using these biomolecules as malignancy-related diagnostic, prognostic, and therapeutic markers. This study aimed to evaluate the potential role of exosomal miRNA as non-invasive biomarkers in DLBCL.

## Material and Methods

### Literature Search

The systematic review was conducted on December 2021 according to the Preferred Reporting Items. To perform this literature review, we searched in the following search engines: PubMed, Scopus, and Web of science to identify all available publications. The search strategy set was adopted utilizing a combination of keywords including: Diffuse large B-cell lymphoma, DLBCL, miRNA, microRNA, miR, exosome, exosomes, exosomal, extracellular vesicles, EVs, and secretome. The full details of the search strategy can be seen in **Table 1**.

**Table 1:** Full details of the search strategy terms.

Terms	Search strategy terms
**Term 1**	(Secretome) AND (Diffuse large B-cell lymphoma OR DLBCL) AND (Prognosis OR survival OR diagnosis OR treatment)
**Term 2**	(Extracellular vesicles OR EVs) AND (Diffuse large B-cell lymphoma OR DLBCL) AND (Prognosis OR survival OR diagnosis OR treatment)
**Term 3**	(Exosome OR exosomes) AND (Diffuse large B-cell lymphoma OR DLBCL) AND (Prognosis OR survival OR diagnosis OR treatment)
**Term 4**	(exosomal miRNA OR exosomal microRNA OR exosomal miR) AND (Diffuse large B-cell lymphoma OR DLBCL) AND (Prognosis OR survival OR diagnosis OR treatment)

### Study Selection

Initially, we excluded duplicate articles and articles without original data. Next, the abstracts were downloaded, and the list was narrowed based on inclusion or exclusion criteria. Subsequently, to verify eligibility, the full texts were evaluated. Further, reference lists of the identified publications were searched for additional relevant studies. The inclusion and exclusion criteria utilized are indicated in **Table 2**. Each eligible criterion was assessed independently by two researchers (S.Y and Z.H) and disagreements were resolved by consensus.

**Table 2:** Inclusion and Exclusion Criteria.

Inclusion Criteria	Exclusion Criteria
Records included Original articles (*in vivo* and *in vitro* studies) Meeting abstracts	Records excluded Duplicated articles Reviews Systematic reviews Editorials
Full-text articles included Original studies must be about DLBCL Original studies must be about exosomal miRNAs Original studies that evaluated the impact of exosomal miRNAs in DLBCL as diagnosis or prognosis biomarkers, or biomarkers that predicted treatment response Original studies that evaluated the molecular pathway that exosomal miRNAs are involved	Full-text articles excluded Duplicate reports EBV+ DLBCL Disease other than DLBCL No analysis on exosomes No analysis on exosomal miRNA No analysis on diagnosis, prognosis, or prediction of treatment response

### Data Extraction and Quality Assessment

Two researchers independently extracted the following information from articles: first author, publication year, type of sample analyzed (tumor tissue, serum, or plasma), sample size (patient versus controls), exosomal miRNAs assessed, number of miRNAs studied, a chemotherapeutic drug, methodology (exosome isolation and miRNA profiling), the origin of exosome and recipient cell (in the case of *in vitro* studies), the list of specific exosomal miRNAs and their molecular characteristic which could be used for diagnosis, prognosis, and therapeutic response prediction in DLBCL.

## Results

### Search Results

**Figure 1:**
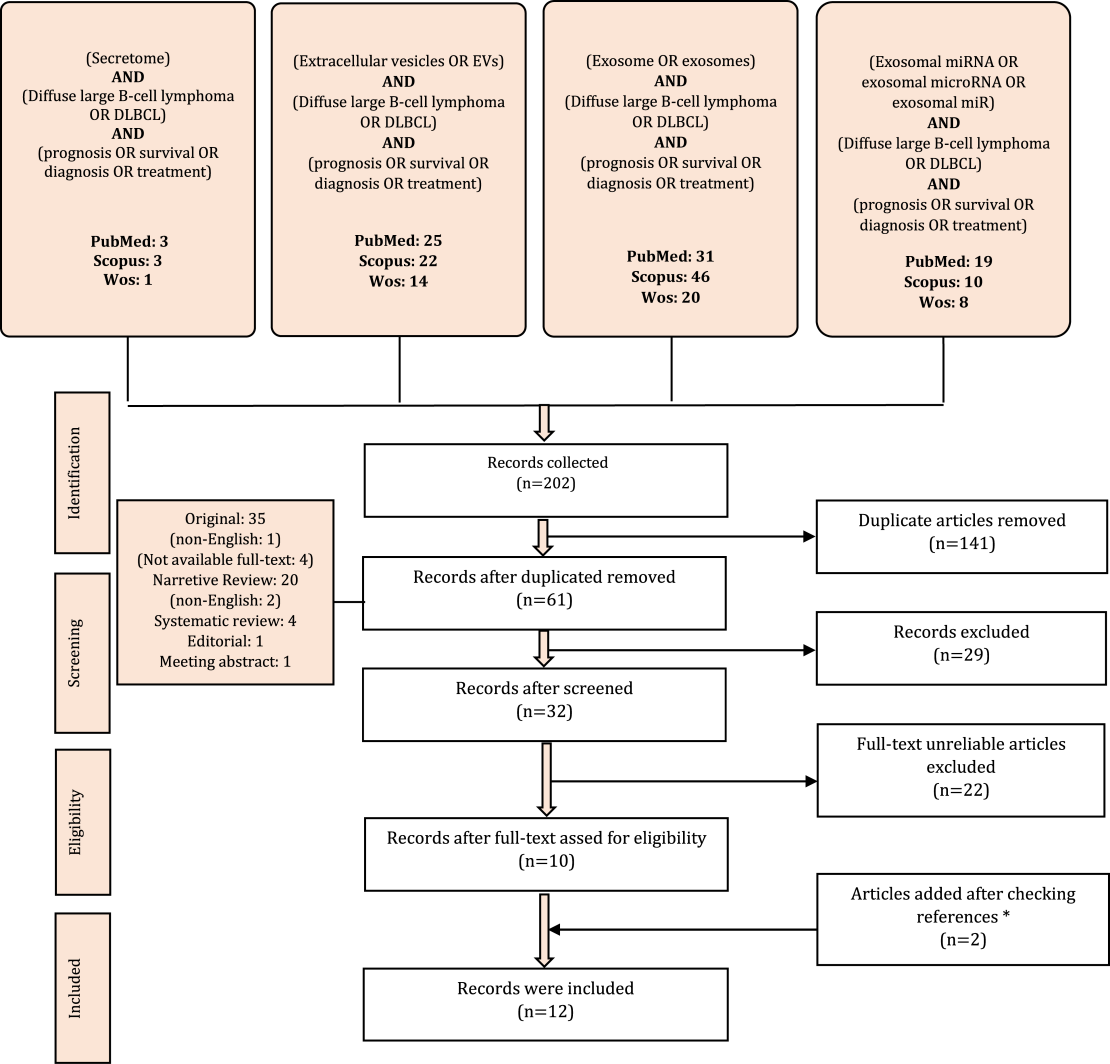
Flowchart of study selection. *We also found two meeting abstracts but did not add them because of the similarity of data with the existing data.

The primary search identified 202 articles that may be related to the potential role of exosomal microRNA in DLBCL (PubMed: 78, Scopus: 81, WoS: 43). Among them, 141 duplicate articles were discarded. Next, after removing 29 records including 20 reviews, 4 systematic reviews, 1 editorial, and 4 articles without accessible full text, 32 records remained. Among them, 22 records were discarded after full-text evaluation (2 articles discussed EVs but have not identified them as exosomes). Additionally, 2 articles were further identified by searching through the reference list of relevant reviews. Finally, 12 articles met the inclusion criteria. The flowchart of the literature study and selection process was shown in **Figure 1**.

### Study Characteristics

Finally, 12 articles investigated the role of exosomal miRNAs in diagnosis, prognosis, and therapeutic response prediction in DLBCL. Of those, 9 articles (75%) discussed miRNAs as diagnostic biomarkers, 4 articles (33.33%) evaluated therapeutic efficacy, 3 articles (25%) provided prognostic biomarkers, 2 articles (16.66%) conducted experiments *in vitro*, and 3 articles (25%) evaluated molecular pathways associated with these exosomal miRNAs in DLBCL (**Tables 3**–**7**).

**Table 3:** Exosomal miRNAs as diagnostic biomarkers in DLBCL.

Study	Exosomal miRNAs	Sample	Source	Exosome isolation	miRNA Profiling	Result
**Exosomal miRNAs with significant differential expressed**						
Rinaldi et al., 2021 (12),	miR-22	27 DLBCL vs. 10 controls	serum	DC	–	Upregulation
Liu et al., 2021 (13),	miR-485-3p	42 DLBCL vs. 31 controls	plasma	ExoEasy Maxi	qRT-PCR	Upregulation
	miR-375-3p miR-107					Downregulation
Cao et al.,2021 (14),*	miR-135a-3p miR-379-5p miR-4476	10 DLBCL vs. 5 controls (Screening stage) 24 DLBCL vs. 24 controls (Training stage) 99 DLBCL vs. 65 controls (Testing stage) 123 DLBCL vs. 89 controls (External testing stage)	serum	ExoQuick	microarray qRT-PCR	Upregulation
	miR-483-3p miR-451a					Downregulation
Caner et al., 2021 (11),	33 miRNAs miR-3960 miR-6089 miR-939-5p others**	20 DLBCL vs. 20 controls	plasma	DC	microarray qRT-PCR	Downregulation
Xiao et al., 2019 (15),	miR-451a	89 DLBCL vs. 48 controls	serum	ExoQuick	qRT-PCR	Downregulation
Khare et al., 2017 (16),	miR-124 miR-532-5p	14 DLBCL vs.20 controls	plasma	DC	RNA seq	Upregulation
	miR-425 miR-145 others***					Downregulation
Inada et al., 2015 (17),	miR-21-5p miR-15a-3p	33 DLBCL vs.22 controls	Serum	DC	qRT-PCR	Upregulation
	miR-181a-5p miR-210-5p					Downregulation
Exosomal miRNAs with insignificant differential expression						
Zare et al., 2019 (18),	miR-146a	48 DLBCL vs. 6 controls	plasma	Exospin	qRT-PCR	NS

*Also, a meeting abstract (19) publish these results with this difference; sample size include: 99 DLBCL vs. 94 controls.

**miR-181b-5p, miR-181d-5p, miR-197-5p, miR-432-5p, miR-595, miR-623, miR-937-5p, miR-1207-5p, miR-1268a, miR-1268b, miR-1307-3p, miR-1587, miR-2861, miR-3656, miR-4481, miR-4632-5p, miR-4701-3p, miR-4707-5p, miR-4721, miR-4725-3p, miR-4741, miR-4763-3p, miR-6127, miR-6724-5p, miR-6803-5p, miR-6840-3p, miR-7108-5p, miR-7845-5p, miR-8069, -miR-8071.

***miR-122, miR-128, miR-141, miR-197, miR-345, miR-424, miR-101, let-7e, miR-222, miR-29c, miR-375, miR-324-5P, miR-135a, miR-379, miR-32, let-7i (Sample size in let-7i analysis: 20 patients vs. 20 controls).

DLBCL, Diffuse large B-cell lymphoma; miR, microRNA; DC, Differential centrifugation; qRT-PCR, quantitative-Real time polymerase chain reaction; RNA seq, RNA sequencing; NS, Non-Significant.

“-”: No information is available.

**Table 4:** Exosomal miRNAs as prognostic biomarkers in DLBCL.

Study	Exosomal miRNAs	Sample	Source	Exosome isolation	miRNA Profiling	Result
Liu et al., 2021 (13),	miR-107	42 DLBCL vs. 31 controls	plasma	ExoEasy Maxi	qRT-PCR	Downregulation: ↓ PFS
Cao et al., 2021 (14),	miR-451a	220 DLBCL (109 DLBCL analyzed for PFS vs. 111 DLBCL analyzed for OS)	serum	ExoQuick	microarray qRT-PCR	Downregulation: ↓ PFS and OS
Feng et al., 2019 (20),	miR-125b-5p miR-99a-5p	116 DLBCL	serum	ExoQuick	RNA seq, qRT-PCR	Upregulation: ↓ PFS

DLBCL, Diffuse large B-cell lymphoma; miR, microRNA; qRT-PCR, quantitative-Real time polymerase chain reaction; PFS, Progression-free survival; OS, Overall survival; ↓, Decreased.

**Table 5:** Exosomal miRNAs as predictor biomarkers for therapeutic efficacy in DLBCL.

Study	Exosomal miRNAs	Sample	Source	Exosome isolation	miRNA Profiling	Result
**Exosomal miRNAs with significant differential expressed**						
Feng et al., 2019 (20),	miR-125b-5p miR-99a-5p	116 DLBCL patients (33 chemoresistant group vs. 83 chemosensitive group)	serum	ExoQuick	RNA seq qRT-PCR	Upregulation in chemoresistant group versus chemosensitive group: ↑ R-CHOP resistance
Zare et al., 2019 (21),	miR-155	48 DLBCL patients (16 refractory/relapsed patients vs. 32 responsive/receiving R-CHOP patients)	plasma	ExoSpin	qRT-PCR	Upregulation in refractory/relapsed patients versus responsive/receiving R-CHOP patients: ↓ response to R-CHOP therapy
	miR-let-7g					Downregulation in patients receiving R-CHOP versus refractory/relapsed patients: ↓ response to R-CHOP therapy
Xiao et al., 2019 (15),	miR-451a	89 DLBCL patients treated with the R-CHOP regimen vs. 48 controls	serum	ExoQuick	qRT-PCR	Upregulation after treatment: biomarkers for prediction response to R-CHOP regimen
**Exosomal miRNAs with insignificant differential expressed**						
Feng et al., 2019 (20),	miR-10a-5p miR-10b-5p	116 DLBCL patients (33 chemoresistant group vs. 83 chemosensitive group)	serum	ExoQuick	RNA seq qRT-PCR	No significant difference between the two groups
Zare et al., 2019 (21),	miR-let-7i	48 DLBCL patients (16 refractory/relapsed patients vs. 32 responsive/receiving R-CHOP patients)	plasma	ExoSpin	qRT-PCR	No significant difference between the two groups
Zare et al., 2019 (18),	miR-146a	48 DLBCL patients (16 refractory patients vs. 32 responsive/receiving R-CHOP patients)	plasma	Exospin	qRT-PCR	No significant difference between the two groups

DLBCL, Diffuse large B-cell lymphoma; miR, microRNA; qRT-PCR, quantitative-Real time polymerase chain reaction; RNA seq, RNA sequencing; R-CHOP, Cyclophosphamide, adriamycin, vincristine, and prednisone, combined with rituximab; ↑, Increased; ↓, Decreased.

**Table 6:** *In vitro* study of exosomal miRNA in DLBCL.

Study	Exosomal miRNAs	Origin of exosome	Recipient cells	Exosome isolation	miRNA Profiling	Result
Zare et al., 2020 (22),	miR-155-5p	DLBCL patients (Responsive and R/R)	NK cells (Healthy donor)	Exospin	qRT-PCR	Upregulation in NK cells: **↓** proliferation and cytotoxicity
	
Zare et al., 2020 (22),	let-7g-5p	DLBCL patients (Responsive)	NK cells (Healthy donor)	Exospin	qRT-PCR	Upregulation in NK cells
Feng et al., 2019 (20),	37miRNAs miR-125b-2-3p miR-99a-5p miR-10b-5p miR-125b-5p miR-146b-3p miR-23a-3p miR-1269a let-7c-5p miR-24-3p miR-novel-chr19 26407	SU-DHL-2 cell SU-DHL-2/R cell	–	ExoQuick	NGS	Upregulation in DLBCL cell lines
Feng et al., 2019 (20),	17 miRNAs miR-122-5p miR-421 miR-144-5p miR-144-3p miR-98-5p miR-577 miR-novel-chr4 51772 miR-novel-chrX 72977 miR-novel-chr20 33917 miR-novel-chrX 73668	SU-DHL-2 cell SU-DHL-2/R cell	–	ExoQuick	NGS	Downregulation in DLBCL cell lines

SUD, LY8 and DUL cells, Diffuse large B-cell lymphoma cell lines; A20 cells, Mouse lymphoma cell line; miR, microRNA; R/R, Refractory/Relapsed; Next generation sequencing; qRT-PCR, quantitative-Real time polymerase chain reaction; ↓, Decreased.

**Table 7:** Molecular pathways associated with exosomal miRNAs in DLBCL.

Study	Exosomal miRNAs	Molecular pathways	Result
Liu et al., 2021 (13),	miR-107	Targeting 14-3-3 η	Downregulation in DLBCL patients: ↓ PFS
Zare et al., 2020 (22),	miR-155-5P	Targeting INPP5D and SOCS-1	Upregulation in NK cells: **↓** proliferation and cytotoxicity
Feng et al., 2019 (20),	37miRNAs miR-125b-2-3p miR-99a-5p miR-10b-5p miR-125b-5p miR-146b-3p miR-23a-3p miR-1269a let-7c-5p miR-24-3p miR-novel-chr19 26407	AMPK signaling* Signaling pathways-regulating pluripotency of stem cells Cellular senescence MicroRNAs in cancer Ras signaling EGFR tyrosine kinase inhibitor resistance mTOR signaling p53 signaling Longevity regulating Autophagy – animal	Upregulation in DLBCL cell lines
Feng et al., 2019 (20),	17 miRNAs miR-122-5p miR-421 miR-144-5p miR-144-3p miR-98-5p miR-577 miR-novel-chr4 51772 miR-novel-chrX 72977 miR-novel-chr20 33917 miR-novel-chrX 73668	Signaling pathways- regulating pluripotency- of stem cells* TGF-beta signaling mTOR signaling Oocyte meiosis p53 signaling Proteoglycans in cancer Pathways in cancer MicroRNAs in cancer Transcriptional- misregulation in cancer cGMP-PKG signaling	Downregulation in DLBCL cell lines

*In the first pathway, all five miRNAs are involved and, in another pathway, some of them are involved.

miR, microRNA; PFS, Progression-free survival; ↓, Decreased.

## Discussion

DLBCL is a clinically heterogeneous lymphoma comprising the most common NHL in adults (23). Diagnosis requires a pathological review of biopsy, which is invasive and can carry limitations for patients (2). Traditional DLBCL risk assessment relies on the International Prognostic Index (IPI) including age, lactate dehydrogenase (LDH) level in serum, disease stage, number of extranodal sites involved, and Eastern Cooperative Oncology Group (ECOG) performance status (24). Of note, the effectiveness of this index has markedly decreased as a result of the use of rituximab during chemotherapy (25). The R-CHOP immunotherapy is the frontline treatment for DLBCL. Even though this type of treatment works in more than half of the cases, about one-third of patients still relapse after treatment (26). On the other hand, DLBCL is an aggressive disease and the survival time of the untreated patient is less than one year (27).

There are many challenges in the DLBCL diagnosis, assessment of patient outcomes, treatment, and prediction of therapeutic response. Therefore, an alternative approach to address these challenges is needed. Based on the importance of miRNAs-derived exosomes, we investigated the literature to evaluate their effectiveness in this field.

miRNAs dysregulation plays a crucial role in the development of different types of cancers by affecting proliferation, apoptosis, cell cycle, angiogenesis, anticancer immune response, and sponge other non-coding RNAs. Considering their huge role in carcinogenesis, the miRNAs panel has great potential for cancer detection, prognosis, and treatment (28). Circulating miRNAs can be incorporated into membrane-coated vesicles, exosomes, or circulate as non-exosomal miRNAs (29). Two unique features of exosomal miRNA, i.e. the remarkable stability and the special function governed by exosomal miRNA (30, 31) render the miRNAs excellent candidates being effective biomarkers.

Evaluation of the implication of exosomal microRNAs profile in diagnosis in DLBCL has been analyzed by nine studies as can be seen in **Table 3**. Among the total miRNAs that have been identified, exosomal mir-451a was found in more than one study (14, 15). Based on the concordance finding of these studies, downregulation of exosomal mir-451a can be a useful biomarker. Of note, both of these two studies examined more than 59 DLBCL samples so their results are more reliable than other investigations (14, 15).

Intriguingly, abnormal expression of three isoforms of exosomal mir-181-5p has been reported in these studies. The results highlighted the down-expression of all isoforms (miR-181a-5p and miR-181b-5p and miR-181d-5p), so further investigation could clarify whether any isoform of exosomal mir-1815p is downregulated in DLBCL (11, 17).

Moreover, the search results revealed that exosomal miR-146a does not show significant differential expression between DLBCL patients and controls (18). It has been demonstrated that an increased level of miR-146a in DLBCL patient tissue compared with individuals with reactive hyperplasia lymphoid nodes (10). Also, studies clearly showed miR-146a upregulation in DLBCL patients (32). In the light that circulating miRNAs can be embedded in exosomes, there is a need for additional studies to confirm exosomal miR-146a diagnostic potential in DLBCL (29).

Regarding the suitability of exosomal miRNAs as prognostic indicators, three articles about four miRNAs were identified (**Table 4**). Among them, elevated levels of exosomal miR-125b-5p and miR-99a-5p were associated with shorter progression-free survival (PFS) while down-expression of exosomal miRNA-107 and miR-451a indicated poor prognosis in DLBCL (13, 14, 20). Despite none of these exosomal miRNAs having been reported in more than one study, analysis on circulating miRNAs can provide evidence that these results are more reliable. For instance, a study identified the prognostic significance of miR-125b-5p in 20 patients with DLBCL (33).

The utility of exosomal miRNAs as predictors for therapeutic efficacy in DLBCL has been identified in four studies (**Table 5**). A total of nine exosomal miRNAs were analyzed: miR-10a-5p, miR-10b-5p, miR-let-7i, and miR-146a did not show significant association with therapeutic efficacy (18, 20, 21). With other miRNAs, although upregulation of exosomal miR-125b-5p, miR-99a-5p, and miR-155 contribute to R-CHOP therapy resistance, downregulation of miR-let-7g was related to it (20, 21). A recent study illustrated high levels of exosomal miR-125b-5p as a chemoresistance-related indicator in DLBCL (20). Additionally, regarding the implication of exosomal miRNAs as predictive biomarkers of response to R-CHOP treatment, it is noteworthy that increasing the level of exosomal miR-451a following therapeutic regimens may implicate exosomal miR-451a as a suitable biomarker for predicting treatment response (15).

We also found two other studies which their finding provided more evidence related to the importance of exosomal miRNAs in DLBCL (**Table 6**). One of these studies evaluated miRNAs expression in DLBCL cell lines (20), while other demonstrated miRNAs transferred through DLBCL-derived exosomes could impacts NK cells function (22).

FInally, we evaluated the molecular pathways associated with exosomal miRNAs (**Table 7**). We hope this molecular characteristic will be used for a therapeutic approach. For example, it has been shown that miR-107 plays a role as a tumor suppressor by targeting 14-3-3 η which results in suppressing oncogenes such as FOXO1, PEPCK, CCND1, P27, BAD, and Bcl-2, so its downregulation is associated with shorter PFS in DLBCL (13). According to this pathway, miR-107/14-3-3 η axis may be a promising therapeutic target in DLBCL (13).

## Conclusion

Taken together, despite the limited number of articles published on this topic, our systematic review demonstrated that exosomal miRNAs not only can be applied for diagnosis and prognosis in DLBCL but also may serve as promising tools for therapeutic interventions and predicting response to treatment. It is hopeful that future investigations will provide more reliable results regarding the clinical significance of exosomal miRNAs in DLBCL.

## Data Availability Statement

The original contributions presented in the study are included in the article/**Supplementary Material**. Further inquiries can be directed to the corresponding authors.

## Author Contributions

SY prepared the manuscript’s backbone and wrote the original draft of the manuscript along with ZH, SK, MI, YL and LB. Z-SC and AG critically revised the manuscript for important intellectual content. All authors have read and approved the final version.

## Conflict of Interest

The authors declare that the research was conducted in the absence of any commercial or financial relationships that could be construed as a potential conflict of interest.

## Publisher’s Note

All claims expressed in this article are solely those of the authors and do not necessarily represent those of their affiliated organizations, or those of the publisher, the editors and the reviewers. Any product that may be evaluated in this article, or claim that may be made by its manufacturer, is not guaranteed or endorsed by the publisher.

## Abbreviations

DLBCL, Diffuse large B-cell lymphoma; NLH, non-Hodgkin lymphoma; R-CHOP, Cyclophosphamide, doxorubicin, vincristine, and prednisone, combination with rituximab; EVs, Extracellular vesicles; sEVs, small extracellular vesicles; lEVs, large extracellular vesicles; TME, Tumor microenvironment; miRNAs, microRNAs; IPI, International Prognostic Index; LDH, Lactase dehydrogenase.
